# The complete mitochondrial genome of an oil-rich microalga *Parachlorella kessleri* TY (Chlorophyta)

**DOI:** 10.1080/23802359.2021.1952118

**Published:** 2021-07-15

**Authors:** Li Ji, Fang-Ru Nan, Shu-Lian Xie, Yuan Li

**Affiliations:** a School of Environmental Science and Engineering, Taiyuan University of Science and Technology, Taiyuan, PR China; b School of Life Science, Shanxi University, Taiyuan, PR China

**Keywords:** *Parachlorella kessleri*, complete mitogenome, genetic information

## Abstract

*Parachlorella kessleri* TY isolated from the lawn soil belongs to Trebouxiophyceae, Chlorophyta. The complete mitogenome of *P. kessleri* sequenced and described. It is a circular duplex molecule 64,744 bp in length consisting of 28 protein-coding genes, 23 transfer RNA (*tRNA*) genes, four ribosomal RNA (*rRNA*) genes, and one putative open reading frames (ORFs). Phylogenetic analysis places *P. kessleri* mitogenome in a branch sister to *Picochlorum sp*., *Lobosphaera incisa*, and *Chloroparvula sp.,* clade in which *Picochlorum* as *P. kessleri* also reported as oil-rich green microalgae.

*Parachlorella* is a genus of green algae, belonging to the Trebouxiophyceae, Chlorophyta. It was established based on the 18S rDNA and ITS sequences (Krienitz et al. 2004) and only three species were reported (Gao et al. 2017). *Parachlorella kessleri* Krienitz once was named *Chlorella kessleri*, collected from Algae Culture Collection of University of Gottingen, Germany (Krienitz et al. 2004). It had been reported as highly productive and oil-rich algal strains with strong adaptability in wastewater (Gao et al. 2017, 2021; Lv et al. 2016, 2019). We sequenced the complete mitogenome of *P. kessleri* in this study, which would facilitate the researches of the phylogeography and phylogenetic relationship of Chlorophyta.

The algae cells of this study were isolated from the lawn soil in a park (37°54′N, 112°33′E), Taiyuan, Shanxi Province, China, and cultivated under axenic conditions (Gao et al. 2017). Voucher specimens (No. SAS2013TY02) were deposited in the herbarium of Shanxi University (SXU). The mitogenome was sequenced by the Illumina Hiseq 2500 technology with 150 bp paired-end library, 350 bp insertion fragments. The Illumina-generated reads were assembled with SPAdes version 3.8.2 (Russia) (Bankevich et al. 2012). Totally 7,314,167 clean reads were obtained for assembly and the sequence coverage depth was 85.2.

Annotation was conducted with Unipro UGENE for initial open reading frame (ORF) finding and blastp for annotation of protein-coding sequences (CDS) (Okonechnikov et al. 2012). Large and small subunits of ribosomal RNA (*rRNA*) were identified using BLASTn with published red algal rRNAs as queries, and transfer RNAs (*tRNA*) and tmRNA were identified using the tRNAscan-SE Search Server (http://lowelab.ucsc.edu/tRNAscan-SE/). The mitochondrial genome of *P. kessleri* was circular and 64,744 bp in length (GenBank accession No. MW533543), consisting of 28 protein-coding genes, 23 *tRNA* genes, four *rRNA* genes, and one putative ORF, which had homologs in other green algal mtDNAs. The protein-coding genes included nine for ribosomal proteins, 10 for NAD(P)H-quinone oxidoreductases (nad), four for ATP synthases, two for cob, one for cox, one for tatC, and one hypothetical protein gene. The overall base composition of the mitochondrial genome was 34.22% for A, 29.05% for T, 14.71% for G, and 22.03% for C. The G-C content was 48.56%, higher than other fresh water green algae (Hu et al. 2020).

For phylogenetic analysis, the data available in the NCBI GenBank database were used. The sequences were analyzed using Modeltest version 3.7 (Spain) to determine the best-fitting models of sequence evolution (Posada and Buckley 2004). Maximum likelihood (ML), neighbor joining (NJ), and Bayesian inference (BI) trees were constructed based on orthologous sequences extracted from aligned data sets comprising sequences of *P. kessleri* and other strains. The orthologous sequences of the mitochondrial genomes were extracted by customized Perl scripts. For the three alignments, topologies recovered based on the ML algorithm are shown in Figure 1, with NJ and Bayesian posterior support values indicated. Phylogenetic analysis of the *P. kessleri* mitogenome placed it as a branch sister to *Picochlorum sp*., *Lobosphaera incisa*, and *Chloroparvula sp.* clade (Figure 1). Some phylogenetic relationships in Figure 1 still lack enough sequence data to support. It is clear that additional sampling of genus *Parachlorella* species will be needed to further clarify the phylogeny and plasticity of the genus.

**Figure 1.**
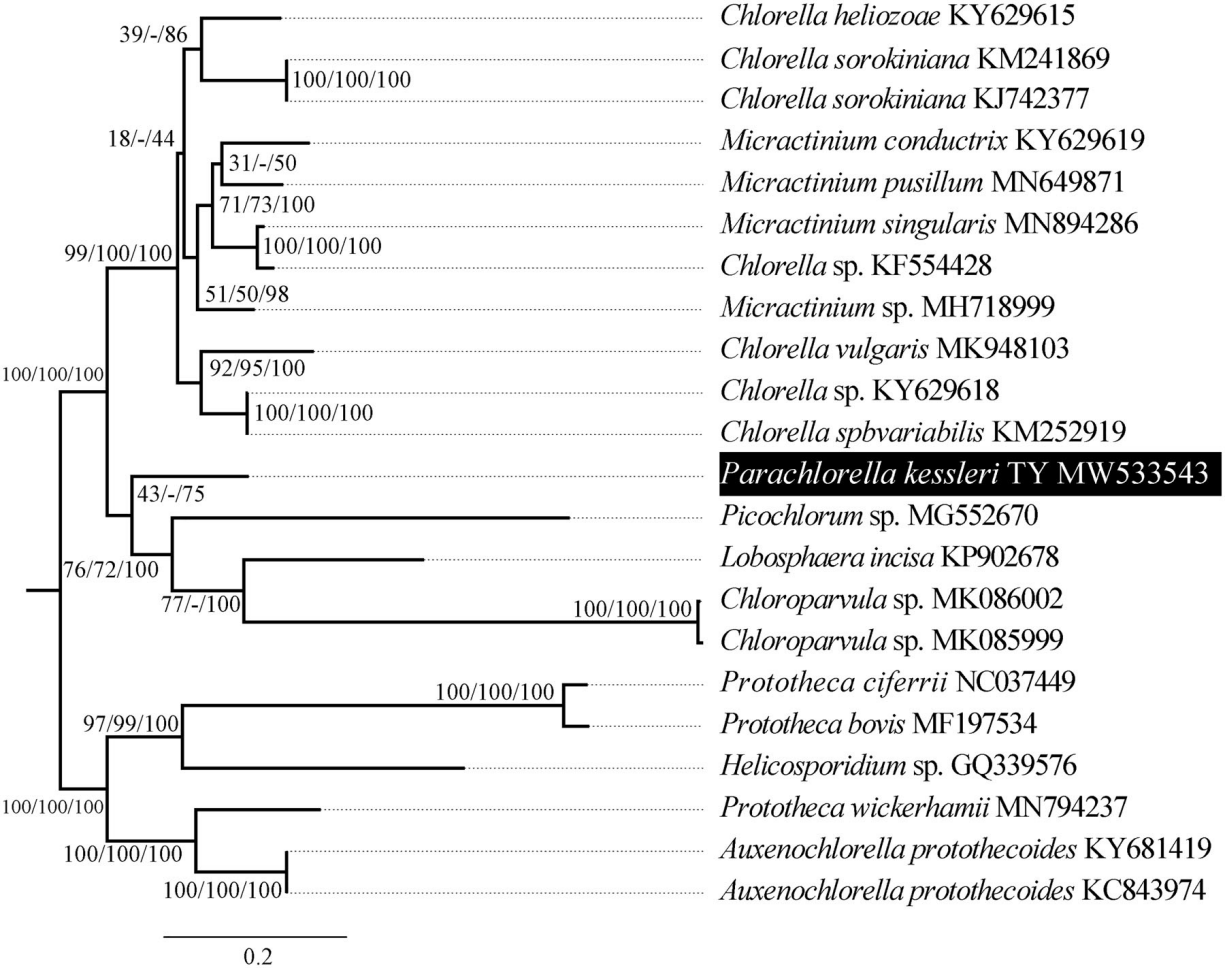
The trees are built according to orthologous sequences extracted from the complete mitogenomes of *Parachlorella kessleri* TY and other species. Support values for individual branches are given as ML bootstrap/NJ bootstrap/Bayesian posterior probability.

## Data Availability

The genome sequence data that support the findings of this study are openly available in GenBank of NCBI at (https://www.ncbi.nlm.nih.gov/) under the accession no. MW533543. The associated BioProject, SRA, and Bio-Sample numbers are PRJN A717157, SUB9362193, and SAMN18478955, respectively.
